# Electrical probing of field-driven cascading quantized transitions of skyrmion cluster states in MnSi nanowires

**DOI:** 10.1038/ncomms8637

**Published:** 2015-07-06

**Authors:** Haifeng Du, Dong Liang, Chiming Jin, Lingyao Kong, Matthew J. Stolt, Wei Ning, Jiyong Yang, Ying Xing, Jian Wang, Renchao Che, Jiadong Zang, Song Jin, Yuheng Zhang, Mingliang Tian

**Affiliations:** 1 High Magnetic Field Laboratory, Chinese Academy of Science (CAS), Hefei, Anhui Province 230031, China.; 2 Department of Chemistry, University of Wisconsin—Madison, 1101 University Avenue, Madison, Wisconsin 53706, USA.; 3 Institute of Fluid Physics, China Academy of Engineering Physics, Mianyang, Sichuan Province 621900, China.; 4 International Center for Quantum Materials, School of Physics, Peking University, Beijing 100871, China.; 5 Advanced Materials Laboratory, Fudan University, Shanghai 200433, China.; 6 Institute for Quantum Matter and Department of Physics and Astronomy, Johns Hopkins University, Baltimore, Maryland 21218, USA.; 7 Department of Physics and Materials Science Program, University of New Hampshire, Durham, New Hampshire 03824, USA.; 8 Collaborative Innovation Center of Advanced Microstructures, Nanjing, Jiangsu Province 210093, China.

## Abstract

Magnetic skyrmions are topologically stable whirlpool-like spin textures that offer great promise as information carriers for future spintronic devices. To enable such applications, particular attention has been focused on the properties of skyrmions in highly confined geometries such as one-dimensional nanowires. Hitherto, it is still experimentally unclear what happens when the width of the nanowire is comparable to that of a single skyrmion. Here, we achieve this by measuring the magnetoresistance in ultra-narrow MnSi nanowires. We observe quantized jumps in magnetoresistance versus magnetic field curves. By tracking the size dependence of the jump number, we infer that skyrmions are assembled into cluster states with a tunable number of skyrmions, in agreement with the Monte Carlo simulations. Our results enable an electric reading of the number of skyrmions in the cluster states, thus laying a solid foundation to realize skyrmion-based memory devices.

## Abstract (web summary)

Future magnetic memory technologies may be based on the manipulation of skyrmions, nanoscale topologically stable circulating magnetization textures arising in chiral magnets. Here, the authors explore the behaviour of skyrmion clusters in nanowires with widths comparable to the single-skyrmion scale.

## Graphical abstract

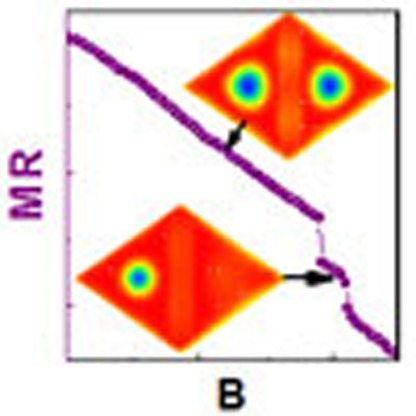

The emergence of topological phenomena and topological materials has attracted growing interests in condensed matter physics^1^. The interplay between topology and geometry not only deepens our physical understanding, but also provides new routes to device designs that incorporate novel physics. In magnetic materials, a notable example of a topologically stable object is the skyrmion, a swirl-like spin texture that carries quantized topological charge^2^,^3^,^4^,^5^,^6^,^7^,^8^,^9^. Skyrmions have been realized in several helimagnets with the non-centrosymmetric B20 crystal structure, such as MnSi, FeGe and Fe_*x*_Co_1−*x*_Si^2^,^5^,^6^. The peculiar twists of the magnetization within the skyrmion originate from the competition between the chiral Dzyaloshinskii–Moriya (DM) interaction and the ferromagnetic (FM) exchange interaction. The ratio of these two interactions determines the skyrmion size, which is typically on the order of 5–100 nm, and can be continuously tuned by doping^10^. Moreover, recent experiments found that skyrmions can be manipulated by electric currents with a current density several orders of magnitude lower than that needed to drive ferromagnetic domain walls^4^,^11^. Although these properties are advantageous for high-density data storage applications with low dissipation, most skyrmions in bulk samples or two-dimensional films reported in literatures have condensed into lattices^6^, which makes the single bit operation impossible. Experimental investigations of epitaxial MnSi/Si(111) films in strong in-plane magnetic field have discovered distorted skyrmion strings^12^, wherein the strain-induced magnetic anisotropy by the Si substrate is crucial for the stabilization of spin textures, and skyrmions are still aligned in one dimension. Although recent study enables an electric reading of the number of turns in the helical structure in MnSi thin films^13^, there is a lack in the detection of creation and annihilation of individual skyrmions in devices by an electric probe that can be more readily integrated into conventional electronic architectures^14^.

Single-crystal nanowires (NWs) of the compounds with the B20 structure provide a valuable insight into this issue^15^. Skyrmion states have been identified in B20 MnSi NWs by Lorentz transmission electron microscopy^16^ and longitudinal magnetoresistance (MR) measurements^17^. However, the widths of the NWs (*d*>200 nm) in these studies are much larger than the skyrmion lattice constant (*L*_*d*_∼18 nm) for MnSi, implying that the skyrmion state therein still condenses into the lattice as in bulk samples. Fortunately, our recent advances in nanomaterials allow us to synthesize high-quality crystalline MnSi NWs of various diameters down to tens of nanometres comparable to the skyrmion size, and conveniently manipulate the thin NWs (see Supplementary Fig. 1 for detailed descriptions of the technology). It is thus an interesting question whether skyrmions can survive in such narrow NWs and behave distinctly.

In this work, we demonstrate the formation of individual skyrmions in thin MnSi NWs monitored by MR measurements. A skyrmion cluster^18^,^19^, composed of sparsely distributed skyrmions rather than skyrmion lattices, is demonstrated in this confined system under an external magnetic field, **B**, aligned along the axis of the wire and confirmed by Monte Carlo (MC) simulations. The MR(*B*) curves exhibit striking jumps at specific fields that corresponds to the creation or annihilation of individual skyrmions. Moreover, the number of jumps depends on the NW diameter, and eventually disappears both in wider and ultra-narrow NWs. Therefore, the jumps in the MR curves reveal the physical signature of the cascading transition of the skyrmion cluster states, and enable an electric reading of the single skrymion.

## 

### Magnetotransport properties of 40 nm NW

MnSi NWs were synthesized by chemical vapour deposition^15^. A representative scanning electron microscopy image of a single wire with a smooth (111) surface is shown in Fig. 1a. The cross-section of the NW shows a merohedral twinning structure with the (001) twin plane parallel with the <110> growth direction (Fig. 1b). This is a common feature of NWs of non-centrosymmetric B20 compounds, in which the unique (001) twin plane partitions the NW into two parts with opposite handedness^20^. A high-resolution transmission electron microscopy (TEM) image (Fig. 1c), together with previous TEM diffraction results^15^, confirms the perfect B20 crystal structure. MR measurements were carried out by a standard four-probe technique on individual NWs (the device is illustrated in Supplementary Fig. 2). We selected a 40 nm diameter NW, the size of which is comparable to *L*_*d*_∼18 nm, to demonstrate the central results. Both resistance (*R*) versus temperature (*T*) and MR–*T* curves are shown in Fig. 1d, where MR at field, *B*, is defined as MR=[*R*(*B*)−*R*(0 Oe)]/*R*(0 Oe). The residual resistivity ratio of the wire is *R*(300 K)/*R*(5 K)∼7.5. The Curie temperature *T*_c_ of the NW is determined to be ∼29 K from Fig. 1d, which is almost the same as that of bulk MnSi (29.5 K). The room temperature resistivity, *ρ*≈198 μΩ cm, is quite close to the value of 180 μΩ cm reported for bulk single-crystal MnSi^21^. These results reflect the high quality of the NW*s*.

Figure 2 shows a typical curve of MR as a function of *B* for the 40 nm NW at *T*=14 K. The origin of the MR is ascribed to the coupling of conduction electrons with chiral modulations^22^,^23^. Previous theoretical calculation^24^ and experimental works on MnSi samples^17^,^25^,^26^ have well established that the magnetic scattering of the conduction electrons by the skyrmion phase would lead to two kinks in the MR(*B*) isotherms, which correspond to the lower and upper critical fields driving the system into and out the skyrmion phase. The data were recorded by increasing the field from −8 kOe to 8 kOe. Following this field-sweeping sequence, 
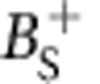
 
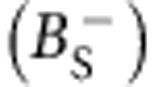
 and 
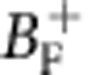
 
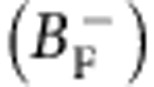
 in the MR(*B*) curve define the transition fields from helical to the skyrmion, and the skyrmion to ferromagnetic phase, respectively^24^,^25^,^26^, or vice versa, where the superscript ‘+ (−)' denotes the positive (negative) branch of the sweeping field. In contrast to the continuous MR(*B*) curves for thick wires (>200 nm; ref. 17), this MR(*B*) curve displays two discontinuous jumps with almost the same amplitudes in the skyrmion phase of *B*_S_*<B<B*_F_. This difference comes from the fact that the skyrmion lattice cannot be supported in the cross-section of such a narrow NW. Instead, a skyrmion cluster state (SC) with sparsely distributed skyrmions is present^18^,^19^. As the skyrmion is a topologically stable spin texture with an apparent particle character, a single skyrmion in the SC cannot be created or destroyed by smooth variation of local moments from other phases. Thus, a field-driven cascading quantized transition is a natural result when the number of skyrmions, *N*_s_, changes one by one under *B*. The maximum number, *N*_max_, in a skyrmion cluster state can be easily estimated^18^,^19^. As discussed above, the presence of a merohedral twin boundary splits the parallelogram cross-section into two triangles^20^. Simple geometry analysis suggests that one skyrmion at most can exist in each triangle, resulting in a total number of *N*_max_=2. This number perfectly matches the number of discontinuous jumps (or drops) in MR.

### Simulated spin textures in 40 nm NW

This interpretation of the MR results is further supported by MC simulations based on the actual sample geometry, in which the 40 nm NW is divided into two merohedral lattices. Details of the model are described in the numerical Methods section. Snapshots of both three-dimensional (3D) and the cross-sectional spin configurations are shown in Fig. 3a–f, where the magnetic field *B* has the unit *B*_R_*=J*/*μ*_B_, with *J* the exchange constant and *μ*_B_ the Bohr magneton. At a low field, a distorted helical order is established with the propagation direction lying in the cross-section and perpendicular to the twin boundary (Fig. 3a). This is different from the conventional bulk sample, where the propagation is along the <111> direction due to weak crystal anisotropy^2^. This difference comes from the presence of the twin boundary, at which the DM interaction vanishes and only ferromagnetic exchange interaction survives. Therefore ferromagnetic ordering is persistent along the twin boundary plane, and modulation along the NW is prohibited. When the field *B/B*_R_ is increased above a threshold, the skyrmion cluster appears (Fig. 3c). Due to the geometric confinement, only one skyrmion is allowed in each merohedral domain and the skyrmions are aligned along the NW, forming two skyrmion tubes. At the NW boundary, spins align parallel to the boundary due to the DM interaction and missing spins near the boundary^18^,^19^,^27^. Consequently, the swirling direction at the boundary of each domain is opposite to that of the skyrmion within, and each skyrmion tries to sit at the centre of each domain owing to the repulsion from the edge states^7^,^8^. Notice that before entering skyrmion cluster state, the system undergoes an intermediated state (Fig. 3b), characterized by forming half-skyrmions in the interior of the NW. As a result, this state weakens the abrupt transition from the helical state into the skyrmion phase, being consistent with the unapparent transition field *B*_S_ in Fig. 2. With further increase of the field strength, one skyrmion disappears, leaving a mixed state of the 3D modulation in one domain and a skyrmion tube in the other domain (Fig. 3d). Although in the simulations, the geometries of the two domains in the NW are identical except for different chiralities, the ferromagnetic exchange at the twin boundary gives rise to certain correlations between two domains. Our result shows that the formation of 3D modulations in one domain can stabilize the remaining skyrmion in the other, so that two skyrmions do not disappear simultaneously. In real samples, the sizes of the two twins are not exactly identical, which may also lead to the difference in the corresponding critical fields. At an even larger field, the remaining skyrmion transforms into the fully polarized ferromagnetic state (Fig. 3f) via distorted conical (C) phase in a tiny magnetic field interval (Fig. 3e), while swirling along the boundary persists (Fig. 3f). The phase diagram for these states is shown in Fig. 3g. This two-step destruction of the SC state corresponds to the two jumps observed in the MR(*B*) curve.

### The size dependence of magnetotransport properties

The resistance jumps are persistent at various temperatures, as shown in Fig. 4a. However, they gradually evolve into kinks at higher temperatures, because thermal fluctuations smear out the topological transitions. Although the kinks become indistinct above 19 K, a closer examination of the dMR/d*B* data (Fig. 4b) still shows two peaks between *B*_S_ and *B*_F_, indicating the survival of the two-skyrmion cluster. Symmetric behaviours are shown in both positive and negative branches of the sweeping fields at high temperatures. However, when the temperature drops <15 K, the magnetization kinetics plays an increasingly important role, as indicated by the reappearance of the conical phase, shown by kinks instead of jumps observed at *B*_F_ in the negative field branches^17^. This conical phase survives in a narrow window sandwiched between *B*_F_ and *B*_C_ shown in Fig. 4a.

Figure 4c shows the *T*–*B* phase diagram constructed from transport measurements, in which the solid symbols are derived from *B*_S_, *B*_C_ and *B*_F_ in the MR(*B*) curves and the open symbols come from the transition temperature *T*_S1_ and *T*_S2_ in the MR(*T*) curves at fixed *B* (Supplementary Fig. 3). It is found that skyrmion clusters with different *N*_s_ are stabilized widely in the *T–B* plane, ranging from the *T*_c_ down to 5 K on the positive field branch, while eclipsed by the conical state in a narrow window at low temperatures on the negative branch. The saturation field *B*_F_ is lower than that previously reported (6 kOe at low temperatures) for bulk MnSi crystals^21^ and thick MnSi wires^17^.

To further understand the importance of NWs size and geometric confinement, we systematically investigated the transport properties of NWs with different diameters (Supplementary Fig. 4). Figure 5a–c show typical MR curves for three NWs with diameters of 80, 55 and 20 nm at representative temperatures. The corresponding dMR(*B*)/d*B* data are simultaneously plotted as grey dotted lines to identify the phase transitions clearly. More discontinuous jumps are observed at a thicker NW with a diameter of 55 nm. A detailed analysis clearly shows three additional jumps with similar jump height in the interval *B*_S_*<B<B*_F_, as labelled in Fig. 5b. A similar numerical calculation shows that only two skyrmions can exist in each triangle divided by the twin boundary, and therefore *N*_max_=4, which perfectly matches the number of jumps (Supplementary Fig. 5). Comparing Fig. 5b and Fig. 4a, we see that the thinner the NW is, the more prominent the discontinuous jumps are. This can be understood phenomenologically from the anisotropic MR^28^, which obeys *ρ*(*θ*)=*ρ*_0_–*ρ*_A_ cos^2^*θ*, where *θ* is the angle between local moments and the cross-section, and the residual resistance *ρ*_0_ is much larger than the anisotropic MR *ρ*_A_. The presence of a skyrmion leads to the spatial modulation of local moments, average over which gives the MR of the wire. It can be easily shown that the resistance change on placement a single skyrmion is given by 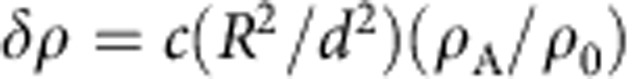, where *R* is the skyrmion radius and *c* is a dimensionless quantity of unity order. Therefore the larger the sample size is, the smaller the jump height will be, as shown in Fig. 5b. We also notice that the jumps in the negative branches are more obvious than those in the positive ones, reinforcing the important role that the magnetization kinetics plays. The observed exceptional data points in the jump region may originate from the emergence of singularities during the topological transition, such as monopoles suggested by the magnetic force microscopy image of bulk Fe_0.5_Co_0.5_Si^29^. In addition, several transitions in the interval 
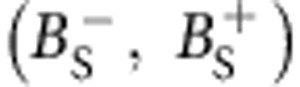
 are also observed. The simulation results suggest these phases are highly relevant to emergence of the elongated skrymion, that is, bimron, the image of which is shown in Supplementary Fig. 5.

For an even thicker NW with a width of 80 nm, the isothermal MR curves display completely different transport behaviour from the narrow NWs, but corroborate the well-established scenario for helimagnets^5^,^17^: the helical phase (H) is stable at low fields (*B<B*_S_) and then transits into the skyrmion state (S) at *B*_S_ via a discontinuous phase transition. At even higher fields (*B>B*_C_), the conical phase appears and eventually turns into the ferromagnetic ordering above *B*_F_. As the NW diameter (*d*∼80 nm) is much larger than the single skyrmion size (*L*_d_∼18 nm), closely packed skyrmions are hosted, leading to a skyrmion lattice, rather than a skyrmion cluster, and the continuous MR*(B)* curve between *B*_S_ and *B*_F_. This is in contrast to the jumps in MR seen in narrow NWs due to the emergence of skyrmion clusters.

As a proof, in the other limit when the NW is extremely narrow, the discontinuous jumps indicating the number of skyrmions should be absent. To this end, a NW with a width of ∼20 nm is examined. The MR follows a smooth curve with two transition fields (marked as *B*_S_ and *B*_C_) in the whole magnetic field region (Fig. 5c). This is expected for such narrow NWs because each small triangular NW fails to host a single skymion (with a size of ∼18 nm). These experimental observations are also reproduced by the MC simulation, as shown in Supplementary Fig. 6, where only helical, distorted conical and field-polarized ferromagnetic states exist. However, the calculated transition fields are much smaller than those inferred from the experimental measurements. On the other hand, compared with the thicker NWs, we observed the high critical temperature and magnetic hysteresis behaviour around *B*_S_ at low temperature. Previous experimental work identified that the critical ordering temperature is strongly affected by finite size effect in NWs^16^. A similar hysteresis was also observed in the easy-plane B20 MnSi/Si(111) film when the magnetic field is applied along the plane^30^. These similarities suggest the important role of finite size effects for such ultra-narrow wires. It has been extensively discussed that the change of lattice structure at the crystal boundary or surface contributes an additional magnetic anisotropy or interfacial DM interaction^31^. Because of the large surface-to-volume ratio in the ultra-narrow NW, we expect these additional interactions to play a significant role in determining the magnetic phases and magnetization process. Nevertheless, based on the MR data and our simple parameter-free model, it is not possible to conclusively decide which magnetic structures are responsible for the field-driven transitions in the MR data. Therefore, based on the numerical results, we only suggest possible helical and distorted conical phases separated by *B*_S_. This is also supported by the fact that the MR curve appears to be similar to that observed in bulk MnSi at low temperatures^25^, where only helical, conical and field-polarized ferromagnetic states exist. Discussions about the additional surface interactions are beyond the scope of this work.

On the basis of the MR data for these NWs at temperatures below *T*_c_ (see the detailed data in Supplementary Fig. 4), we can summarize the *T–B* phase diagrams for NWs with different diameters, as shown in Fig. 5d–f. For *d*∼20 nm, the critical field *B*_F_ is much larger than that in bulk MnSi (Fig. 5f). Importantly, as the size of the NW increases, a skyrmion cluster phase emerges. At *d*∼40 nm, which is about two times larger than *L*_d_, for example, the skyrmion cluster state is dominant while the conical state appears in a tiny *T–B* window on the negative branches at low temperatures (Fig. 4c). As the NW diameter further increases, individual skyrmions are closely packed into a lattice (Fig. 5d) through a crossover region (Fig. 5e).

## Discussion

We have demonstrated the presence of skyrmion cluster states in confined MnSi NWs with diameters comparable to single-skyrmion domain size. The maximum number of skyrmions within this cluster is determined by the dimension of the NW cross-sections, and the number of skyrmions can be controlled by external magnetic fields and revealed by quantized jumps in the MR curves. These results not only reveal new physics of the skyrmion cluster states in confined geometries, but also can guide the development of skyrmion-based memory devices in which the individual skyrmions could be utilized for multibit memory cells.

## Methods

### Device fabrication

All MnSi nanowires (NWs) used in this work were synthesized by chemical vapour deposition using MnCl_2_ precursor onto silicon substrates^14^. To fabricate NW devices for magnetotransport measurements, a single NW was picked up from the original silicon substrates and transferred onto a new clean Si substrate coated with 300 nm silicon oxide by three-axis hanging joystick oil hydraulic micromanipulators under an optical microscope, where a home-made tip was used to pick up the NW (See Supplementary Fig. 1). Four Pt or W electrodes were patterned onto an individual NW using focused-ion beam techniques with a small beam current of 7.7 or 24 pA, using a FEI Helios Nanolab 600i. Before Pt deposition, the contact areas were milled by ∼5 nm with a beam current of 1.1 pA to remove surface oxide. To avoid contamination of the electrode and destruction of the sample by the Ga ion source, a 200 nm thick PMMA resist was used to cover the rest of the samples between the electrodes by regular e-beam lithography technique before the fabrication of the electrodes (See Supplementary Fig. 2).

### Measurement methods

Standard four-probe transport measurements on individual NWs were carried out in a Physical Property Measurement System (Quantum Design Inc.) with a 16 T superconducting magnet. For the transport measurements, to obtain good data with high signal-to-noise ratio, large currents of 30, 10, 5 and 2 μA were used to measure the 80, 55, 40 and 20 nm NWs, respectively. Transport data under small currents were also measured to make sure that the effects of joule heating on the experimental results due to large current density were negligible. All MR*(B)* data were recorded by increasing *B* from −8 kOe to 8 kOe. The sweeping fields corresponded to the properties of NWs under field cooling (FC). We also recorded the initial MR curves, reflecting the properties of NWs under zero FC (by cooling the NWs to the defined temperature from far >*T*_c_, for example, ∼50 K, in zero magnetic field before recording the MR data). However, this difference between ZFC and FC does not significantly affect the final results (Supplementary Fig. 7).

### MC simulations

To support the experimental observations, MC simulations were performed to get a clear picture of the spin configurations. A generally accepted Hamiltonian for chiral magnets including direct and indirect magnetic coupling with constants *A* and *D*, magnetization **m**, and external field energy is written as^29^,^32^


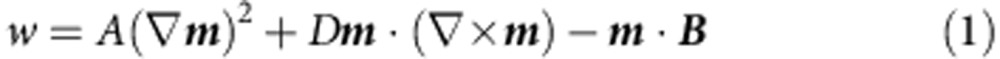


For the MC simulation, the 40 nm NW is divided into two discrete blocks with unit magnetization **S**, then, a 3D lattice Hamiltionian corresponding to equation (1) is written as


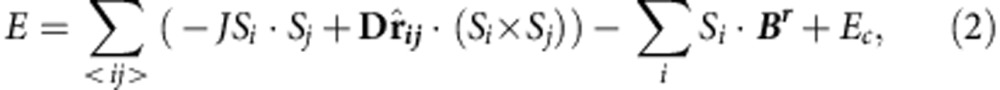


where *J* denotes the ferromagnetic exchange coupling constant; 
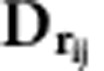
 is DM interaction vector with 
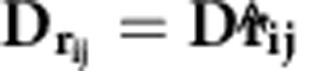
 and 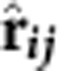 is the vector pointing along site i and j; <ij> denotes the nearest spins. **B**^**r**^ stands for the reduced magnetic field. The constructed model is schematically illustrated in Supplementary Fig. 8.

In the discrete model, both direct and DM exchange in an inhomogeneous state will have slightly different energy depending on their orientation with respect to the underlying discrete bond orientation of the crystal lattice. Contrary to the isotropic continuous model in equation (1), the discrete model in equation (2) used in the MC simulations leads to the anisotropy energy. To smear out this effect, a corrected term *E*_*c*_ is added as^29^


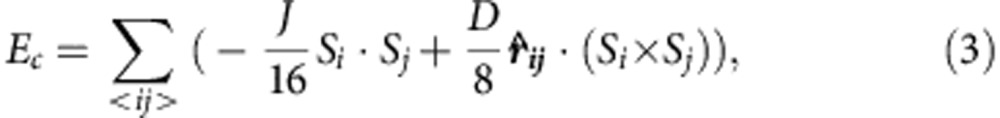


where <*<*ij*>>* represents the next nearest spins. The ratio *D/J* was chosen to be consistent with the value adopted in ref. 29, where the wavelengths of *d*=10 lattice constants were used.

Concerning the real geometry, open and periodic boundary conditions were adopted in the cross-sectional plane of the NW and the long axis, respectively, and a boundary forming the same NW geometry as the experimental merohedral twin plane, which divides the NW into two equal parts with opposite chirality^32^. Thus, the sign of the coefficient *D* is opposite in the two domains. According to the previous Lorentz TEM observations in another B20 compounds FeGe, the inversion of the lattice chirality (handedness) of the B20 structure across certain grain boundaries would lead to the invariance of the sign of the spin–orbit interaction within FeGe. It was also observed that the spins around the boundary are almost parallel with the grain boundary. This observation implies that the ferromagnetic interaction would play a dominant role in the narrow transition region where the inversion of the lattice chirality (handedness) occurs, yielding the nearly parallel spin arrangements in the thin region of crystal boundaries^33^. Thus, the value of *D* is set to zero in the boundaries^20^. A high temperature annealing metropolis algorithm is used to obtain the equilibrium spin configurations. At each temperature, the system is allowed to relax towards equilibrium for the first 10^5^ MC steps and thermal averages are calculated over the subsequent 10^5^ steps.

## Additional information

**How to cite this article:** Du, H. *et al*. Electrical probing of field-driven cascading quantized transitions of skyrmion cluster states in MnSi nanowires. *Nat. Commun.* 6:7637 doi: 10.1038/ncomms8637 (2015).

## Supplementary Material

Supplementary Information Supplementary Figures 1-8.

## Figures and Tables

**Figure 1:**
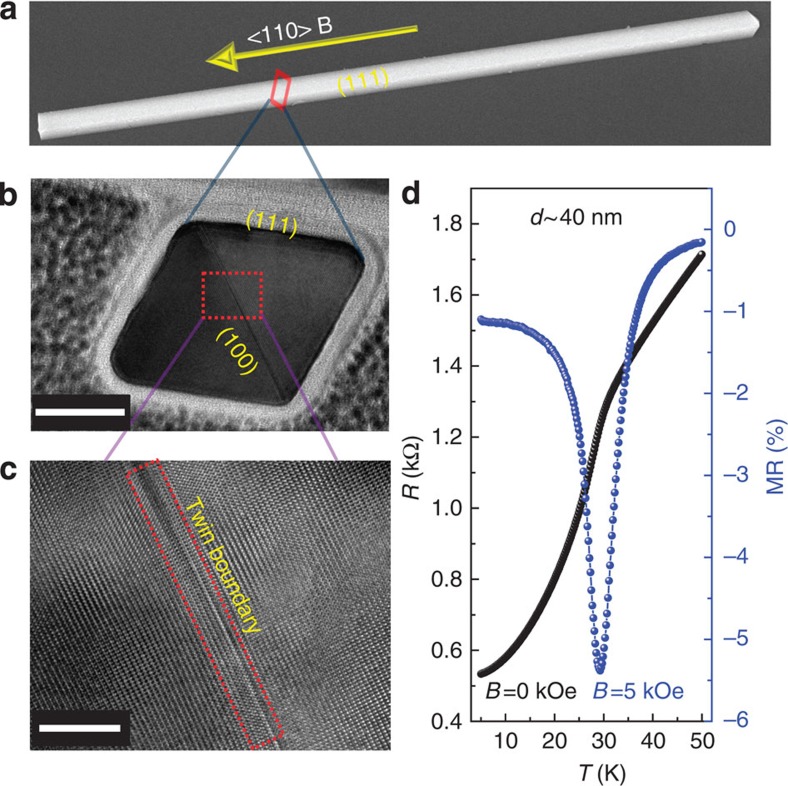
Crystal morphology and transport properties of a MnSi NW. (**a**) A typical scanning electron microscopy (SEM) image of a MnSi NW with a smooth (111) surface and <110> growth direction. (**b**) A cross-sectional TEM image of a NW with a merohedral twin boundary, where the (001) twin plane is parallel with the <110> growth direction. (**c**) High-resolution TEM image of the cross-section at the twin boundary. (**d**) Temperature dependence of the resistance (black) and magnetoresistance (MR; coloured dots) at 0 and 5 kOe, showing a *T*_c_ of 29 K. Scale bars, 20 and 6 nm in **b** and **c**, respectively.

**Figure 2:**
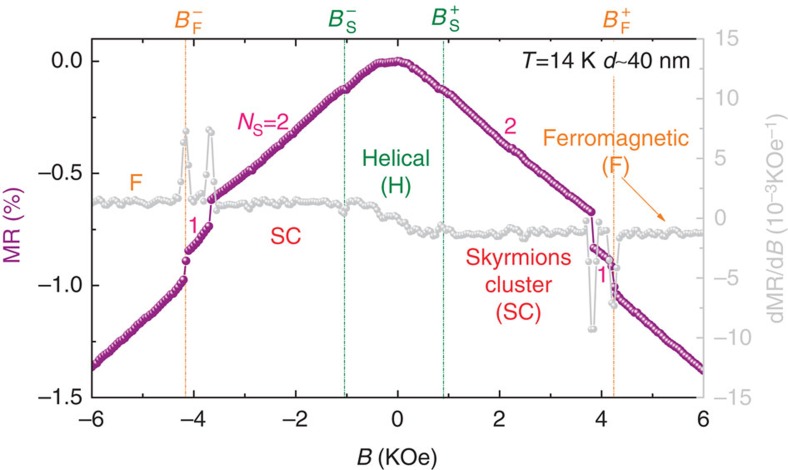
Variation of spin configurations with magnetic fields for a 40 nm NW. MR (coloured dots) and dMR/d*B* (light grey dots) versus the magnetic field *B*. The measurements were carried out by increasing the field from −8 kOe to 8 kOe. *N*_S_ represents the number of skyrmions in the skyrmion cluster states. The transition fields, extracted from the dMR/d*B* data, at positive and negative sweeping magnetic field branches are denoted by the superscripts ‘+' and ‘−', respectively. H, SC and F stand for the helical, skyrmion clusters and ferromagnetic phases, respectively.

**Figure 3:**
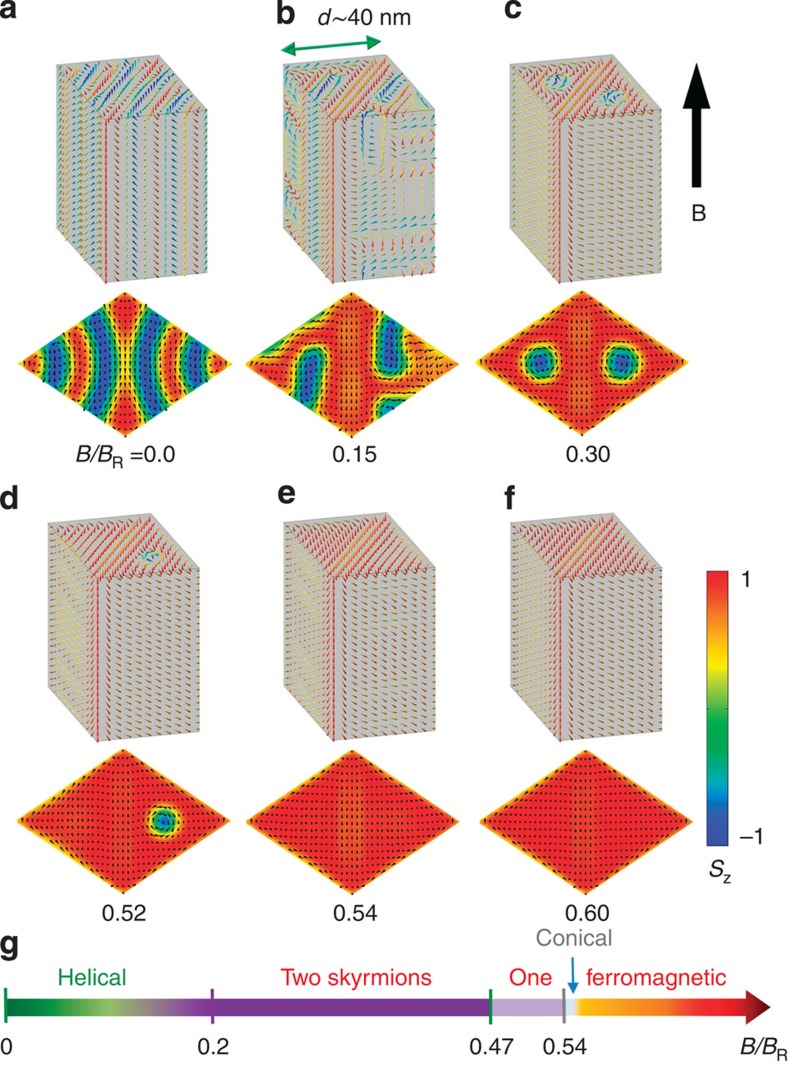
Calculated spin configurations for the typical states in the 40 nm NW. (**a**) Distorted helical; (**b**) intermediated state with meron in the interior of the NW; (**c**) two skyrmions; (**d**) one skyrmion; (**e**) distorted conical phase or 3D modulations; (**f**) field-polarized ferromagnetic state) and the corresponding cross-sections of these states. (**g**) The phase diagram in *B* space, where *B*_R_*=J/μ*_*B*_ is the unit of magnetic field in the MC simulations.

**Figure 4:**
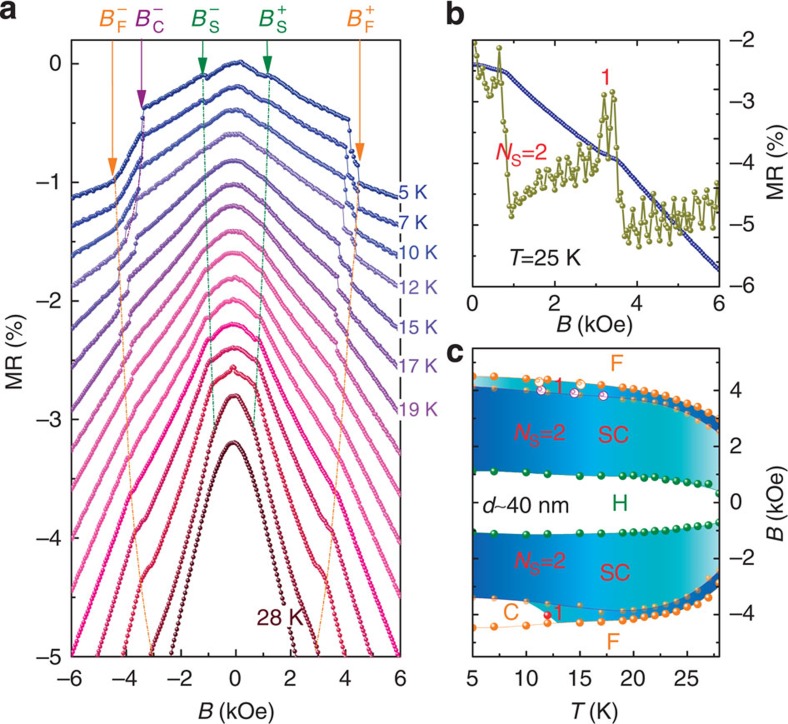
Transport properties and *T–B* phase diagram of a 40 nm MnSi NW. (**a**) MR at various temperatures below *T*_c_. Three transition fields are marked by different colours. The data are shifted for clarity and the temperature is increased in 1 K steps from 19 to 28 K. (**b**) MR and dMR/d*B* as a function of *B* at a high temperature T=25 K, where two peaks in the dMR/d*B* indicate the persistence of two individual skyrmions. (**c**) Phase diagram inferred from MR data. Solid and open symbols represent data obtained from MR(*B*) and MR(*T*), respectively. *N*_S_ stands for the number of skyrmions in the cluster. Individual skyrmions survive in a large *T–B* window down to the lowest measured temperature (5 K) in the positive magnetic branches. A small pocket labeled by C is the conical phase.

**Figure 5:**
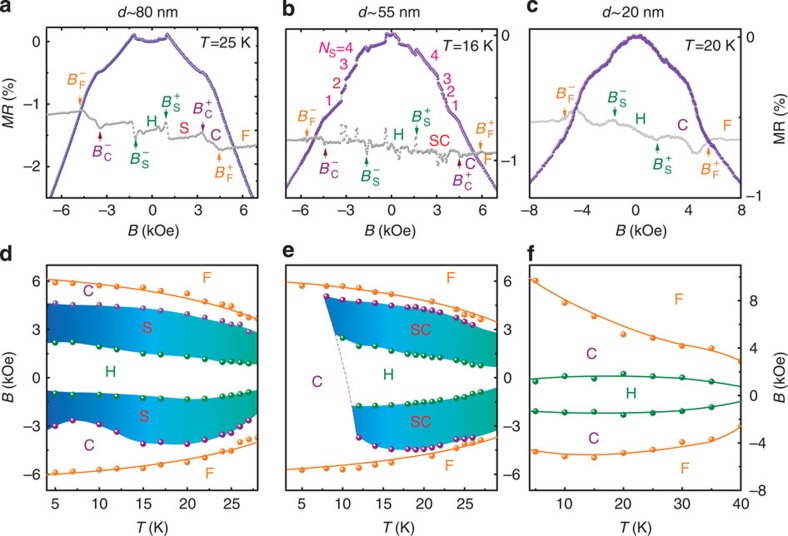
The NW diameter dependence of the MR behaviors and the skyrmion phase diagrams of temperature *T* and magnetic field *B*. (**a**–**c**) MR as a function of *B* for three NWs with different diameters (coloured dots; (**a**) 80 nm; (**b**) 55 nm; (**c**) 20 nm). The transition fields are extracted from the corresponding dMR/d*B* data (light grey dots). (**d**,**e**,**f**) The phase diagrams obtained from the MR(*B*) data. S stands for the skyrmion lattice state. The coloured regions indicate the skyrmion states.
