# Supplementary material for: Electrical probing of field-driven cascading quantized transitions of skyrmion cluster states in MnSi nanowires
Source: Nat Commun. 2015 Jul 6;6:7637. doi: 10.1038/ncomms8637 (PMC4506500; doi:10.1038/ncomms8637)
Supplement: Supplementary Information — Supplementary Figures 1-8. [file ncomms8637-s1.pdf]

Supplementary Figures

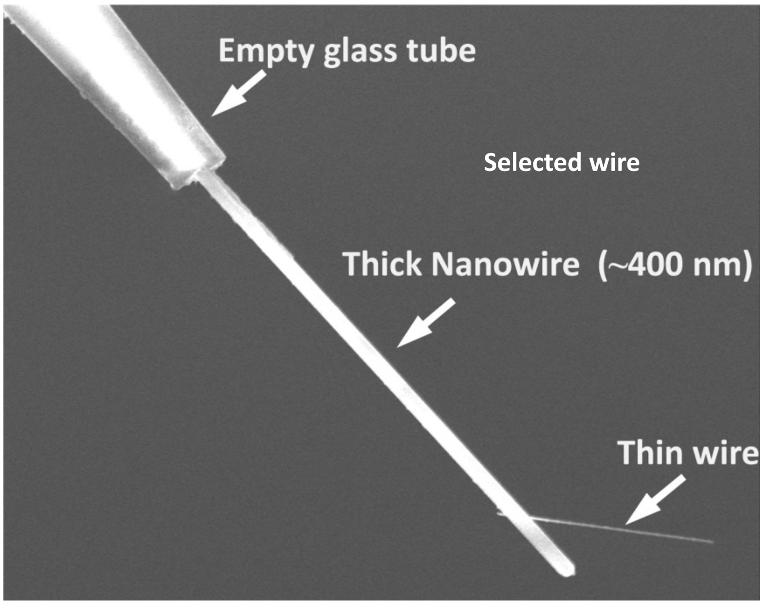

Supplementary Figure 1 | Microtip for the transfer of NWs. A thick NW is inserted into a hollow glass tube. This wire is used as a new tip instead of the capillary glass tube for picking up the NW samples. In this way, the success rate for selecting the desired NW is significantly improved. A thin NW is stuck on the thick NW at its tip.

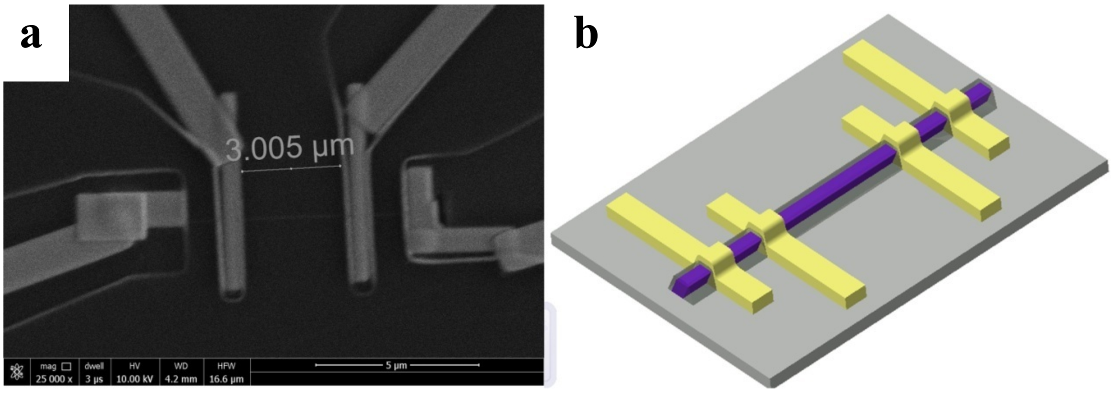

Supplementary Figure 2 | Four-probe electrical device for NWs transport measurements. a, A typical scanning electron microscopy (SEM) image of a MnSi NW device. We first used e-beam lithographic technique to expose the PMMA resist in the intended areas of the electrodes. After the developing process, we got the well-defined e-beam pattern on the NW. In order to achieve good ohmic contact with negligible contact resistance to the MnSi NWs, we deposited four Pt electrodes by

focused ion beam (FIB) technique at the areas defined by e-beam, the rest of the NW samples between the electrodes are still covered by 200 nm thick PMMA electron beam lithography resist for protection of the NW. **b**, A schematic illustration of the device with four electrodes (yellow stripe) and MnSi wire (purple).

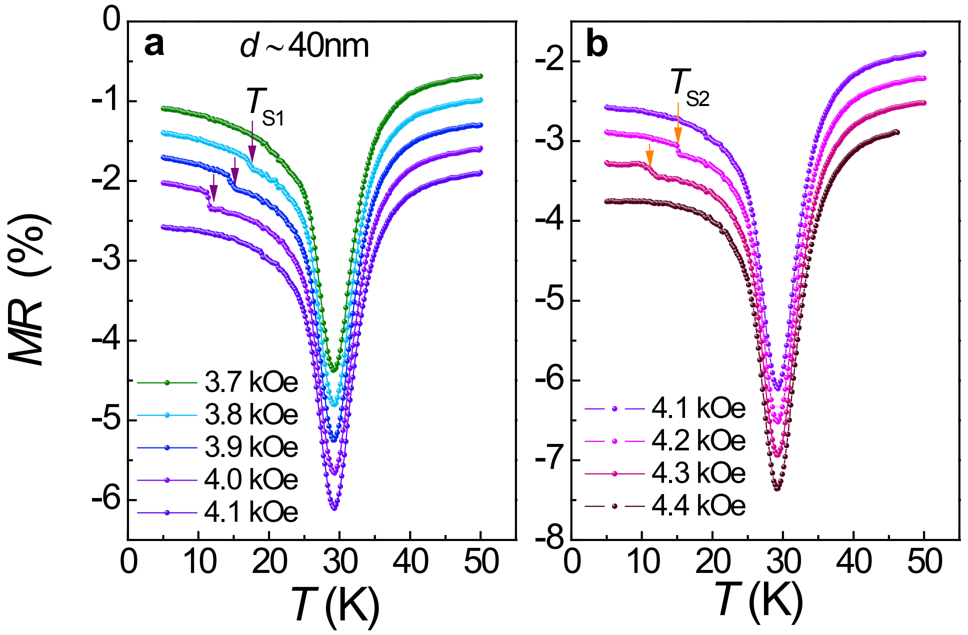

Supplementary Figure 3 | Temperature dependence of the *MR* for the 40 *nm* NW under different magnetic fields. a, The magnetic fields from 3.7 kOe to 4.1 kOe; **b,** The magnetic fields from 4.1 kOe to 4.4 kOe. A deep valley around T_c is seen due to the suppression of carrier scattering from spin fluctuations. Below T_c, a more complex *MR* behavior that sensitively varies with the increase of *B* is seen between 3.7 kOe and 4.4 kOe. A comparative analysis of the *MR* vs. *T* and *MR* vs. *B* data allows us to conclude that T_{S1} corresponds to the lower critical temperature from two skyrmions into one, while T_{S2} corresponds to the transition temperature from one skyrmion into the ferromagnetic state. All data are shifted for clarity.

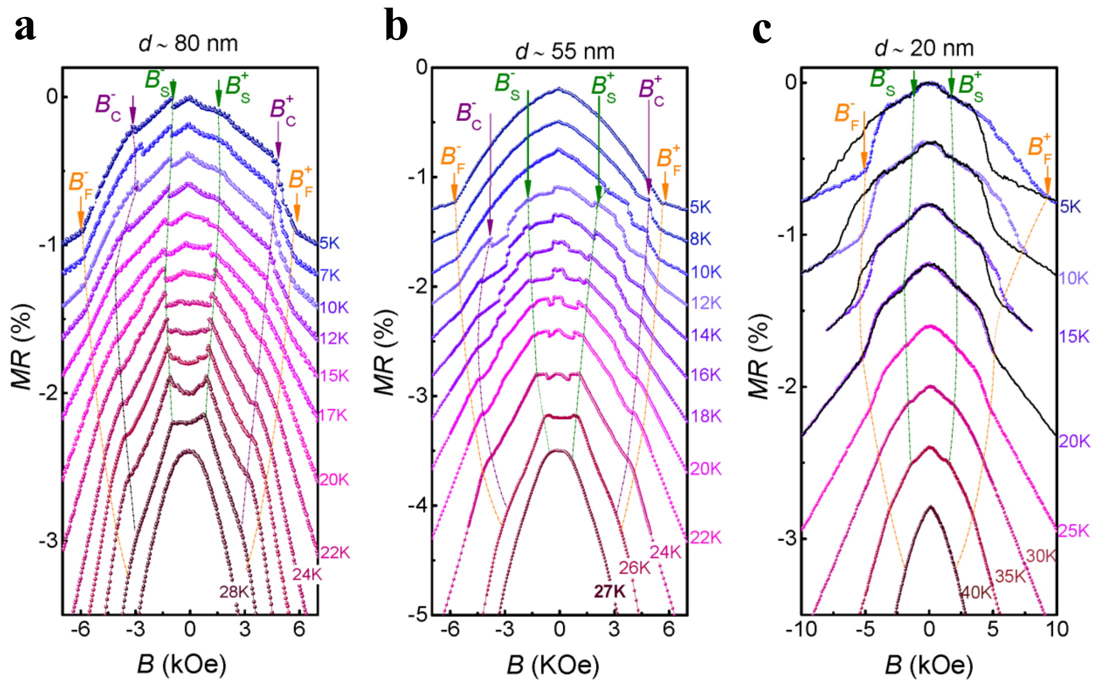

Supplementary Figure 4 | *MR* as a function of *B* for three NWs with different diameters (a, 80 nm; b, 55 nm; c, 20 nm) below T_c. The transition fields B_S, B_C and B_F are defined the same as in the main text. The dotted lines mark the phase transitions for the guiding the eyes. For the 80 nm NW, *MR* curves show continuous change in the skyrmion state between B_S and B_C, while discontinuous ones are observed for the 55 nm NW. Further decreasing the size of the NW to 20 nm caused the skyrmion state to disappear. All data are shifted for clarity. The black lines in **c** show the data collected when the field was swept from positive to negative and significant hysteresis loops appear below about ~ 20 K.

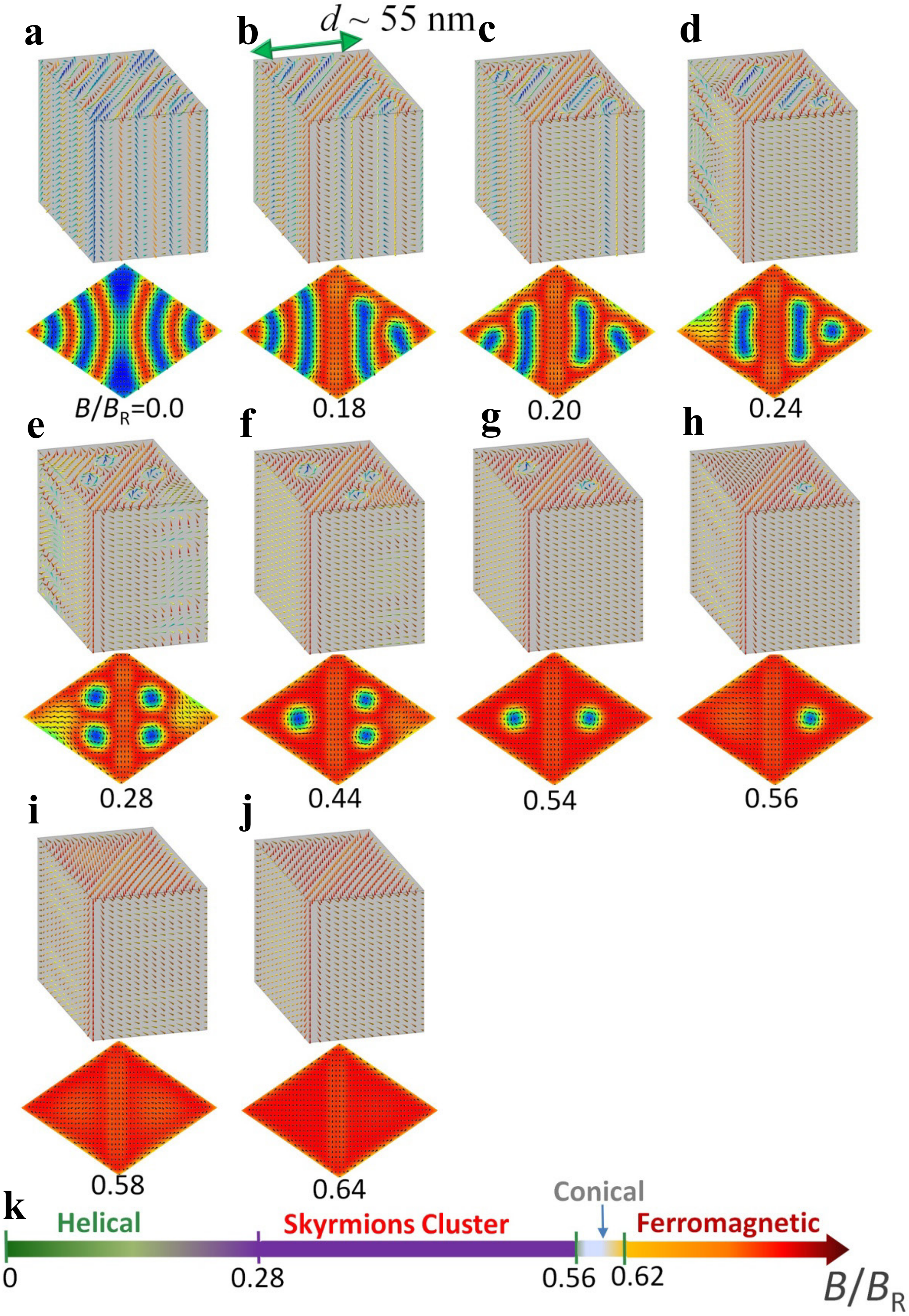

Supplementary Figure 5 | Spin configurations for the magnetic states in the 55 nm NW. **a**, distorted helical state. **b-d**, intermediated state with the mixture of bimeron and skyrmions in the interior of the NW. The bimeron and skyrmion should respond to the observed jumps in the interval (B_S^+,B_S^-) shown in Figure 5b. **e**, four skyrmions. **f**, three skyrmions. **g**, two skyrmions; **h**, one skyrmions. **i**, distorted conical phase (3D modulation); **j**: field-polarized ferromagnetic state)**.** **k**, The phase diagram in B space.

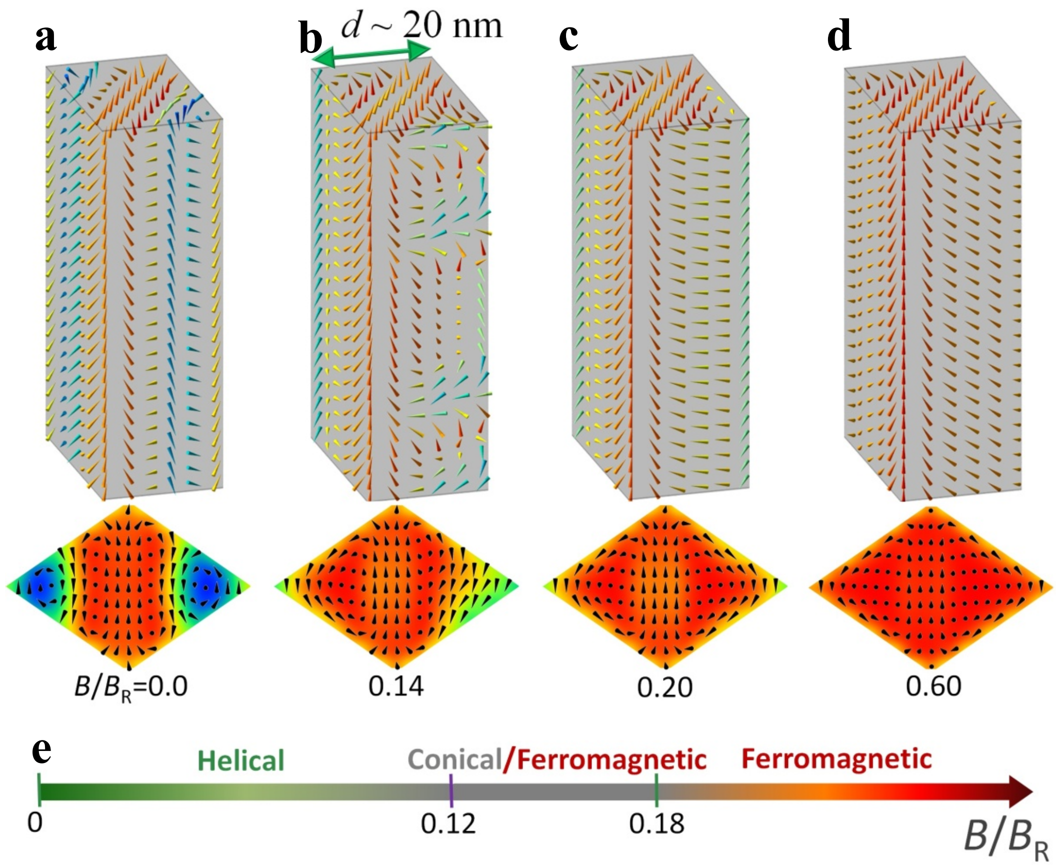

Supplementary Figure 6 | Spin configurations for the magnetic states in the 20 nm NW. a: distorted helical state. **b**, distorted conical phase (3D modulations); **c-d**: field-polarized ferromagnetic state)**. e**, The phase diagram in *B* space.

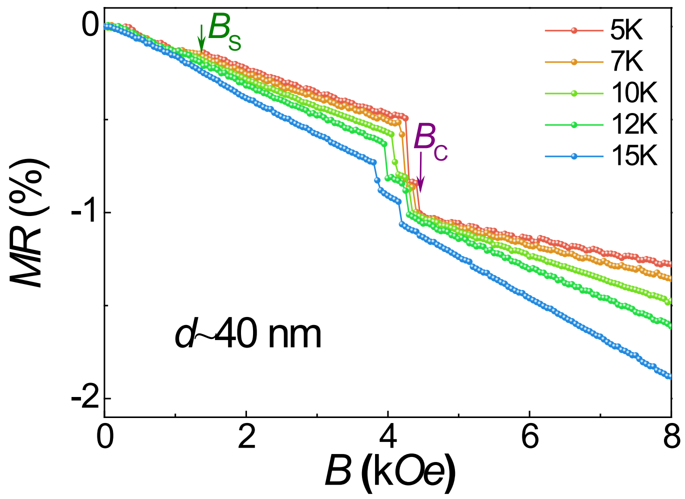

Supplementary Figure 7 | The magnetic field dependence of the *MR* for the 40 nm NW under ZFC. To obtain the data under ZFC, we raised the temperature far above T_c (e.g., ~ 50 K) in zero magnetic field for each measurement, and then cooled the NWs to the defined temperature in zero magnetic field before recording the *MR*

data from zero field to high fields. It is noted that above 15 K, the curves under FC and ZFC are the same. Below 10 K, the *MR* curves after ZFC show a close agreement with that in the positive branch under FC.

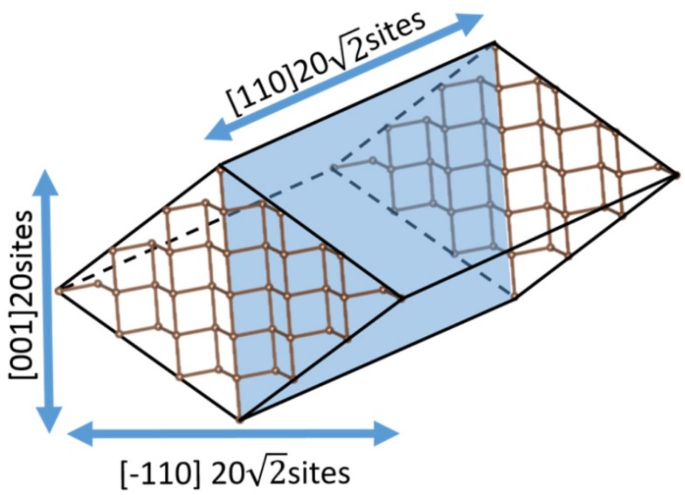

Supplementary Figure 8 | The orientation of the simple cubic lattice used in our MC simulations. The blue shaded areas mark the twin plane.
